# Mr. Solly on the Physiology and Pathology of the Human Brain

**Published:** 1848-01-01

**Authors:** 


Art. II.—*The Human Brain: its Structure, Physiology, and Diseases, with a Description of the Typical Forms of Brain in the Animal Kingdom.* By Samuel Solly, Esq., F.R.S., Senior Assistant Surgeon to St. Thomas's Hospital. Second Edition. London: Longman, 1847. Pp. 673. 8vo.

Five-and-twenty or thirty years ago, the medical student had no standard work on physiology to refer to. The truths of this science were scattered up and down the pages of practical writers, or else they were interwoven throughout the text of works on anatomy, or were, at the best, but only introduced as a sort of prolegomena to the course of anatomical lectures, upon the opening of each session at the different schools of medicine in the metropolis. At all events, there was no treatise on physiology in vogue of a decidedly indigenous character; for we recollect with what pleasure we first took up Richerand's Physiology, translated from the French by Copland, and Blumenbach's, with its very entertaining and instructive notes by Elliotson; or how eagerly we turned out what we were in search of from the clever Physiological Proems in Mason Good's "Study of Medicine." This season of scientific destitution has, happily for us, however, passed away, and been followed by an abundance not less satisfactory than the harvest that has so providentially crowned the autumn of the past year.

The two Hunters certainly took the lead in this country. But the museum of the College of Surgeons, with its magnificent host of preparations that live or repose along its walls, was, as it were, a sealed book to the mass of medical men; while the celebrated Treatise on the Blood, with its apothegms, and half-told general rules, and universal laws, was couched in a language so peculiarly obscure, that the routine practitioner, unused to recondite studies, or the intricacies of grammatical construction, disregarded it, as utterly beyond the reach of his capacity, and to all intents and purposes valueless to him as a book of practical utility. The amount of John Hunter's knowledge of the nervous system was small enough, and the extent of it is summarily drawn up by the reviewer of Mr. Palmer's edition of Hunter's works, in the following words:—"Hunter's view was so especially comprised within the range of the vegetative or organic functions,—the formation of blood, and its distribution throughout the body,—that he had not time to look up to the particular functions of the brain as the nervous centre, nor even to glance at the wide relations of the ganglionic system."*

To his brother, Dr. W. Hunter's, work on the Gravid Uterus, and the splendid collection of preparations and casts in illustration of it, it is quite superfluous for us to do more than to allude. But however excellent they may have been in themselves,—and, indeed, their excellence is such, that they remain to this day a work *sui generis* and complete, as

* Med. Gaz. 1838, vol. xxii. p. 649. In the same periodical there is a digest of the Treatise on the Blood, which pretends to afford a clue to the scope and aim of Hunter's thoughts. The writer says:—"Upon the properties and functions of the nerves themselves, Hunter is very brief. I know of only two passages where he directly notices their use or influence."—*Med. Gaz.* 1828, vol. iii. p. 596.

far as they go,—they are, nevertheless, far from meeting the views now taken, and the new opinions, or rather demonstrative conclusions, put forward or entertained by the present generation of pathologists. We have in our possession an authentic manuscript copy of Dr. W. Hunter's Lectures on Midwifery, which pretend to give the anatomy and physiology of the subject of which he is treating, but from which we cannot extract a single statement of the slightest weight as far as regards the functions of the cerebro-spinal system of nerves.

Such was the condition of physiology in this country at the time we mention. In the meanwhile, however, the Continent had been giving birth to physiologists of no ordinary growth and stature, who had, with a master mind and at a giant's pace, been making great strides into the hitherto almost unexplored regions of the brain and its interesting radiation of nerves. Their freedom of thought and accuracy of investigation, coupled together with the startling novelty of their discoveries, astonished the retired English dissector in particular, or shocked the received prejudices and comfortable religious assurances of the lay public in general. To say nothing of Baron Cuvier—"medio dux agmine, tota vertice supra est"—we refer to the writings of Meckel, Tiedeman, Magendie, Cruveilhier, Flourens, and others, with peculiar feelings of delight and gratitude, for the atmosphere of truth they seemed to breathe around us, as well as for the extent of intellectual horizon they so unexpectedly unfolded to our view. We were in a new land. Like Rinaldo, beneath the charms of Armida, we were spell-bound, conscious of a sudden increase of knowledge, and alive to our entrance on a pathway through the fields of science to which we had hitherto been entire strangers. But the direction of our journey was not altogether continental; for Sir C. Bell, Mayo, and Marshall Hall recalled us to our native shores, where our attention was arrested by the discovery of the motor and sensory nerves, the posterior roots and ganglions of the spinal cord; and the exquisitely beautiful explanation of the reflex functions of the spinal column, unquestionably due to the natural talent, genius, and industry of our own countryman, Marshall Hall himself. From thence we proceeded to the fibrous structure of the brain; and this pursuit turned our inquiry away from England, and directed it once more to the Continent, and Germany in particular; where Gall,* generally known in Great Britain only so far back as twenty or five-and-twenty years ago, had already begun to assert and demonstrate the fibrous structure of the brain as early as the beginning of the present century. Considering how completely time has verified the principles, and attested the dissections of Gall, it is now astonishing to reflect with how much vituperation he was assailed in this country. A few of the orthodox literary journals of the day—we refer particularly to those published in 1815 and 1817,

* Professor Owen says:—"The *fibrous structure* of the brain, the discovery of which, though due to Coiter, as early as 1573, has sometimes been attributed to Reil and Gall, is displayed by Hunter in preparations made to show the fact, (Nos. 1335, 1336,) and is expressly mentioned in the description of the anatomy of the whale."—Palmer's edition, Hunter's Works, vol. iv., preface, p. xvi. Although we forget the particular date, we remember perfectly well when Gall presented himself to Sir A. Cooper, at St. Thomas's Hospital. Of course, he was quite a lion, or, in the estimation of those who disapproved of his "*doctrines*," a venomous animal.

joined successively in decrying both Gall and Spurzheim's discoveries or dissections as quackery, absurdities, trash, and nonsense !* And these periodicals, that uttered their sinister omens from the dim niche of partial knowledge, were but the re-echoes of private prejudice and ignorance, which have, like birds of twilight, vanished before the noonday of the age. We were once, while hospital students, in company with the late Venerable Archdeacon Nares, the reputed editor of the "British Critic." "I hope," said the Archdeacon, addressing us, "they do not teach phrenology at your hospital?" We honestly answered, they did not. "Because," continued the venerable dignitary of the establishment, "phrenology is a sad thing, as it leads to scepticism, and a disbelief in the truths of revealed religion!"† We treasured up this invaluable hint with boyish fidelity, and for a length of time afterwards piously refrained from approaching the excommunicated topic of phrenology. Yet in our later days, no one can have been more seriously addicted to phrenology than ourselves; nor are we aware of having sustained thereby any diminution in our creed, nor of having relied with less affectionate devotion on the Supreme Author of our being. "On a subject thus obscure in all its parts," says Dr. Holland, the most dispassionate of its advocates, "and where our actual knowledge is still limited to detached facts or presumptions, there is enough to justify the opinion being kept before us, as one of the outlines to which future observations may apply."‡ We shall have occasion, by and by, to notice Reil and Foville's works on the brain, as well as Grainger's; or if we do not specifically notice them ourselves, it is because such ample justice has been done them by Mr. Solly, the last, though by no means the least, among the intelligent labourers in this department of science.

Mr. Solly's work is the first that can be fairly considered as a complete essay on the brain. The dissection of the nerves was formerly a very dry and stupid affair, that of the brain was a mere tissue of hard names without meaning; while the dissection of the spinal cord was, with the exception of enumerating its thirty-two pairs of nerves, a very cursory piece of anatomy indeed. To a practised dissector of the "*old school*," the present mode of tracing the fibres of the brain must inevitably cause a thorough revolution of his ideas. He must make up his mind to go through that very difficult and perplexing process of upsetting all the knowledge he has hitherto gathered and arranged,—of wiping out the greater part of it entirely from the tablet of his memory,—and of beginning altogether anew in acquiring fresh notions, constructing another set of images, and informing himself with doctrines and principles, most provokingly at variance with those that he has heretofore been accustomed to uphold with all the veneration and respect due to established truths. For, instead of slicing off the brain from above downwards, he must now dissect it from below upwards; instead of examining surfaces, and exploring ventricles, he must now trace out the fibres which form the

* Dr. Elliotson's "Physiology," 5th edition, 1835, p. 383.

† No one could esteem the Archdeacon Nares more highly than ourselves, and the above anecdote is given merely to show what was the prevailing temper of the day, even among the best informed.

‡ "Notes and Reflections," by Dr. Holland, p. 511.

corpora quadrigemina, constitute the visible striæ of the transverse commissure, or diverge into the convoluted expansion of the hemispherical ganglion.

"Every honest and erudite anatomist," says Mr. Solly, in his preface (p. xi.), "must acknowledge that we are indebted mainly to Gall and Spurzheim for the improvements which have been made in our mode of dissecting the brain. For my own part, I most cheerfully acknowledge that the interest which I derived from the lectures of Dr. Spurzheim, at St. Thomas's Hospital, about the years 1822 and 1823, has been the exciting cause of all the labour which, for above twenty years, I have at intervals devoted to this subject. I believe that to Mr. Green, in his 'Dissector's Manual,' is due the honour of having first given to the English student an abstract of Gall and Spurzheim's method of dissecting the brain. Mr. South, in his edition, enlarged it considerably."

The leading idea of Mr. Solly's work is, that a greater development of the nervous system bears a direct ratio to a greater development of the mind; that, all things being considered, the larger the mind, the greater the brain,—the finer the precision of its delicate organization, the higher the intelligence of which it is the outstanding organ, and which, in the order of God's providence, it is supremely appointed to enunciate and subserve: that among the visible miracles of this our miraculous existence, the brain is the instrument of thought, and that the manifestation of thought corresponds exactly with the more or less perfect development of the nervous centres. Startling as this proposition may sound in some ears, even in this day of science thus far advanced, it is, nevertheless, the truth, grounded upon fact, nor can it be rejected, unless we are prepared to reject the evidence of our senses, and to refuse the data of our common understanding. The common numerals of common arithmetic no more surely represent the sum total for which they are made to stand, than the amount of respectable witnesses subpœnaed to give their special evidence on this point, can fail in attesting this.

From the maggot that leaps from a nut as we crack it on our plate after dinner, and the caterpillar that eats up the leaves of our favourite convolvulus in the garden,—from the fish that cleaves the green, translucent wave, and the bird that wings the breeze of incense-breathing morn,—from the lion that roams the desert wild, and the horse that tramps the battle field, or prances before the lady's equipage,*—up to Man, the master of them all, there is one all-pervading nervous system, progressively diminishing in a downward scale of analytic exhaustion, till it ends in the mere microscopic globule of a brain, by which they all communicate and hold their relative and inter-dependent existences, according to their various forms and needs, and types of organization, function, growth, location, and pursuits.

* The *reins* between the horse's mouth and the coachman's hands are *supplemental nerves* of communication, whereby the horse is endowed with a superior intelligence in the man, and the man is empowered with an additional strength of body in the horse, for the time being. Thus, by the means of the two *occasional* motor and sensory nerves (*the reins*), the superior brain governs the inferior, both animals being thereby rendered one and the same.

The knife of the dexterous dissector may display, and the most powerful lens of the best microscopist may reveal, the amazing intricacies of their minute anatomy, but the lens and the knife only increase the already marvellous intricacies of structure and organ, and only remove us so much the farther from the ultimate point of their elaborate, consentaneous, and multiform vitality. But what we cannot learn by direct investigation, we may arrive at indirectly by reflection, induction, analogy, and comparison, applied to similar and collateral objects. In the wide range of comparative anatomy, the elements of human physiology lie detached, wide apart, and exposed to view, so that, in the more simple manifestations of life, we may discover that which lies concealed from our researches in the more complex structures of the higher organic formations—comparative anatomy being an analysis of human physiology. Each creature, from a polyp up to man, is an entity, but it is an entity becoming evermore simplified under an exhaustive analysis, from man down to a polyp.

Now, if we take Mr. Solly's book for our guide, we may follow out this reasoning in a series of matter of fact details. After some observations on the grey and medullary matter of the brain, he proceeds to comparative anatomy in general, and adopts the five divisions of the animal kingdom, according to Cuvier, Grant, Rudolphi, and Macleay, "named in accordance with the form and arrangement of the nervous system," in which we are presented with a bird's-eye view of the matter of the brain, beginning in an almost indiscernible point, and ascending to the globular brain of man and the higher mammalia.* He begins with the lowest living creatures. First, the intestinal worm, with its microscopic thread of nerves; next, the star-fish, with nervous filaments, and the nodule of a brain. This at once leads him to some of the recondite parts of anatomy; the ganglion of the fifth,—the cineritious matter of the brain, considered as a peculiar organ in itself,—and the truth of the *grey* neurine *generating power*, and the *white conducting* it. Then come the phenomena of life, (of which there is an analytical and synthetical diagram, worthy of attention,) and the history of the ganglionic nerves neatly and concisely narrated. The rudimental brain of the ascaris terminates in a ganglionic centre, which is shown at length in the articulata; and the law of development is carried upwards through the lobster, oyster, (with its supposed power of vision !)† the snail, slug, moth, &c., till the primordial brain is developed almost entirely in insects, and the earliest example of reflex functions produced in the mantilla of the cuttle-fish—the imperfect development of the nervous masses harmonizing with the low or imperfect habitudes of these creatures.

* Plato says—"The gods bound the two divine circulations of the soul in a *spherical body*, in imitation of the circular form of the universe; and this part of the body is what we denominate the head—a most divine member, and the sovereign ruler of our whole corporeal composition."—Taylor's Plato, 4to, vol. ii.; Timæus, p. 516.

† Garner, (Lin. Trans., vol. xvii. part iv. p. 485,) has stated that distinct, though very simple, organs of vision may be observed on the margin of the mantle. It has long been known to fishermen, that the shadow of a boat passing over a bed of oysters will cause them to close their shells; this we can hardly suppose would occur if they were not supplied with some form of the apparatus of vision.—Solly on the Brain, p. 46.

We thence go on ascending through the vertebrata, such as the fishes, amphibia, reptiles, and birds, observing how exactly intelligence and hemispherical ganglia, or grey neurine, increase at the same time *pari passu,* till we are brought to a halt by the mammalia, that important class of animals at the very foot of man himself. The distinction between the placentalia and implacentalia is nicely drawn, and it is curious to remark the difference of intelligence, and also the difference of brain, between animals whose birth is typified by placental or non-placental fœtation. The kangaroo (non-placental) is scarcely above the bird in intellect; but the rabit (placental) has made a sudden step in advance towards the human intellect above the kangaroo; and in the brain of the sheep we find the human brain no longer rudimental, but complete. The porpoise, which nurses its young, and the elephant, which judges for itself, have each of them brains more highly organized, according to the higher intelligence of either animal; and it is in the mammalia, much more plainly than in birds, that we fairly arrive at the conclusion, of mind being in some manner associated with the convoluted surface of the brain, and of the cortical substance, or grey neurine, being the appropriate ganglion or organ of thought and will—called the hemispherical or intelligential ganglion. The reason of the brain's being convoluted is assigned—namely, for the sake of a larger surface being folded and packed up within a smaller compass; accordingly, the deeper the convolutions, the greater the extent of surface packed; the more extensive the surface thus packed, and the deeper the furrows, the more energetic the mind. A classification of animals is attempted, in groups of similar convolutions, which is intended to associate animals of corresponding faculties.

The whole is clever, clearly narrated, and consecutively worked out in a chain of logical induction or actual demonstration. Plates accompany the text, and help to teach by the eye what the mind might happen to misunderstand in mere verbal description. It is the work of an artist, as well as the production of a man of genius.

In the lower animals, the skeleton is external or deciduous, as in the lobster, which draws its claws out of its old shells, as we do our legs out of our boots; but in fishes, and the higher mammalia, as well as in man, the skeleton is internal and permanent. Perhaps the first sketch of anything approaching to the idea of a skeleton is in the shell of the cuttle-fish, or in the more beautiful protective apparatus of the pearly nautilus. But here we again run foul of the "*excommunicated topic*" of phrenology, which stands out in a singularly convincing manner, when we are shown how the exterior hard skull is modelled by the interior soft brain *from within!* It would take up too much time to consider the ready manner in which Mr. Solly seizes every opportunity of explaining human by comparative anatomy: thus the reflections of the arachnoid remind him of the fœtal membranes,—the vascularity of the pia mater, of placental tufts. And so, likewise, of pathology: senile dementia and atrophy of the brain, spina bifida and cerebral pressure, float through the author's mind, as it were, together with his thoughts on the cerebro-spinal fluid. The weight of the human brain is handled in the same dexterous manner, and every turn introduces us to some interesting fact or matter for deeper inquiry or research. But the chief part is yet to come, and this is the fibrous structure of the brain itself, made up of all the fibres of all the nerves from all the different points in every different part of the body: in fact, the brain is to the nerves what a terminus is to a set of diverging railroads, or what Downing-street is to the colonies and dependencies of the British empire, every report being conveyed thither, and every order issued from thence.

But the most striking part remains yet untold, which is, that each nerve is double, made up of a motor and sensory filament, similar, as Dr. Billing, in his First Principles, says, to strands in a rope, or threads in a skein of silk. Each nerve, thus twofold in itself, enters the brain along the sensor and motor tracts, and terminates in the grey neurine, or rather, it passes through the grey neurine, and expands itself in a thin white layer on the convoluted surface, to which it communicates, and from which it receives the orders of volition. So very wonderful is this demonstration (for demonstration it is), that all we can do is to stand still, wrapt in meditation and delight!

We have not yet touched on the lesser brain—the spinal cord—that nervous centre which so accurately unites the instinctive or ganglionic nerves with the voluntary or cerebral, and governs those subsidiary movements which are carried on unconsciously while our attention is engaged on objects far beyond our reach, or actually suspended in sleep. This is a concise explanation of what are called the reflex functions, of which there is a diagram in the table of contents, that will at once convey an idea of it to those whom our words have failed to enlighten. At the risk of being hypercritical, we might make a remark respecting this diagram, that it could have been so drawn out as to represent the decussation of fibres at the pons varolii, as well as the principle of the reflex movements at the same time. And respecting diagrams in general, we may observe, that they are always serviceable, since they partake in a great measure of the character of a mathematical problem, and convince us, that what can be so concisely expressed by signs, is in all probability absolutely true. The highest forms of philosophy are the apothegmatical or axiomatic, the mathematical, and the experimental.*

There is a chapter on the development of the human brain, or rather, on embryology. Those who have given the sanction of their great names, and borne witness, by the extent of their labours, to the justness of the views here put forth (and they rank among them the first scientific names in Europe), regard embryology as a passing comparative anatomy, and comparative anatomy as a permanent embryology.†

* Albertus Magnus (circ. A.D. 1250), a writer scarcely known, and not read at present, was, with the exception of his great disciple, nicknamed the "*silent ox*" (bos mutus), the clearest of writers on analytic philosophy. He divides his inquiries into twenty heads, too long to be here enumerated; but he places comparative anatomy as a subject of the last degree of importance in his list. Modern scholars are agreed that Albert was a man of singular sagacity, and that his treatises on plants, minerals, and animals, prove him to be an observer of the highest order.—*Histoire de St. Thomas d'Aquin, par M. Delécluze.* Paris, 1844. Pp. 105—107.

† On the 11th October, 1831, a lecture was delivered at King's College on Embryology, by that accomplished and talented physician, Dr. Ferguson. It was not printed, we believe. In the above paragraph, perhaps, Dr. F. may track our footsteps in his own snow.

The pathology is so short, that it only leads us to wish for more. Grounded, as it is, upon the best medical and surgical authorities—Abercrombie and Andral, Bright, Burrowes, Copland, Watson, and others—it is presented in a form almost beyond the reach of criticism, as far at least as the matter itself is concerned. The only remarks that can be ventured upon are the judiciousness with which these authorities have been chosen as guides, and the mode in which their steps have been followed. But, in saying this, it is not meant to imply that Mr. Solly himself is no guide—just the contrary; for the experience of an extensive hospital, like that of St. Thomas's, with its ample resources, well-managed wards, and 800 beds, has not been thrown away upon him, neither has he omitted to draw out cases, in illustration or confirmation of his views, at full length. Whatever may be the objection *to daily reports* of cases, both as to their tediousness, and the facility with which they slip the memory, their real value consists in their being statements and records for reference of the most certain kind. A case reported in the form of a narrative cannot, however interesting it may be, fail to receive some colouring from the reporter's imagination and habitual tone of thought; but a dry report of daily symptoms and treatments, with the result, is of the stubborn nature of a first principle or fact, and must be conclusive. Gooch, Morgagni, and Sydenham, are narrators of cases. Andral, and his school, are statistical reporters. They both have their merits; but for all scientific purposes the *dry school* is the best.

Mr. Solly has divided his pathology into four classes—sanguineous (hyperæmia), and exsanguineous (anæmia) of the brain,—convulsive and organic. The great question which Monro Secundus is convicted of being guilty of propounding—namely, that the amount of blood is at all times always the same within the calvarium, seems to be now entirely set at rest by the admirable experiments of Dr. Burrowes on this subject. There can be no doubt (indeed, *we* never doubted it) that the tide of circulation through the head is liable to ebb and flow;—that the flood-tide brings in with it all the plethoric diseases—meningitis, cerebritis, apoplexy, congestive coma, &c., strictly belonging to the antiphlogistic school, with its formidable train of breaching batteries and forlorn hopes; while the ebb-tide, on the contrary, leaves the citadel of the mind open and defenceless to the invasion of fatuity, atrophy, ramollissement, hydrocephalus, asthenic coma, and that frightful phantom, *delirium tremens*. Convulsive diseases are still enveloped in their own obscurity. Their symptoms, indicative as they are of imminent danger to mind and body, are as yet *unpathologized*, and their intimate nature remains unresolved, or else carries us away on the wings of conjecture into the remote regions of the ultimate molecules of the nervous structure. *Post mortem* dissection has served but little to elucidate this perplexing subject—the most practical writers can assign no local habitation to epilepsy, nor is the distinction clearly defined between epilepsy and convulsions in consequence of teething or fracture of the skull. And so likewise as to mania—a nebula scarcely discernible in the "*clear-obscure*" (chiar' oscuro) of the dead-house, or from behind the veil that so mercifully shrouds the chambers of an appropriate asylum. Much might be done on this interesting question, by erasing theory, and recording the ascertained data of morbid anatomy in connexion with lunacy or madness. We have no doubt that the number of these data is exceedingly small. A great many remarks on insanity are interspersed throughout this part of Mr. Solly's work, and show that his thoughts are already bent in this direction. We exhort him to persevere—the field is open before him, and the wards of St. Thomas's are teeming with the richest materials. Every one of his patients is a treatise on disease, and each disease is a monograph on its own pathology. It only requires attentiveness, a mind inclined to observation, and endued with an ardent love of its profession, in order to work out these materials into the happiest results. Organic disease of the encephalon, and that singular form of scrofula, hypertrophy of the brain, close the pathology, and (for the present only, we hope) bring Mr. Solly's labours to a close likewise.

Many diseases of the cerebro-spinal system yet remain almost intact. All those diseases of the head in connexion with primary and secondary disorders of the kidney, which the indefatigable Prout has only touched upon—the fatal coma of ischuria renalis, and cerebral exhaustion, with the phosphatic diathesis. There is, also, tic doloureux from anæmia curable by steel, and the hidden pathology of sciatica and neuralgia in general, which the dogmatic Macculloch forces into his capacious fen of marsh diseases. Add to all these, the functional and organic diseases of the spinal cord or column, which Mr. Solly has elsewhere slightly treated of, but in which no writer has yet shone, except Sir B. Brodie, in the present day, and Hippocrates, still more brightly, 2250 years before him.* The curious subject of dreaming belongs likewise to the pathology of the brain. Aristotle, his scholiast, or commentator, somewhere remarks, that dreams partake of the temperament of the person—that hot blood gives rise to vivid imaginations, and that cold dispositions dream of ice or water. It is important, he adds, that the physician should attend to the kind of dreams, as he may from thence gain some insight into the nature of his patient's malady.

Our paper is diminishing with every line, but our subject is dilating in our hands. We have not yet noticed duality of mind, neither has Mr. Solly, except a brief allusion in his preface to Dr. Wigan's work on this novel and interesting question. Dr. Holland, in his Notes and Reflections, has a chapter on it. Cicero's head was unequal, one side being larger than the other.† Neither have we yet dwelt with sufficient minuteness on the importance of the convolutions as a manifestation of mind. When the foldings and furrows are deep, it renders the head long, and, as Mr. Solly remarks, the vulgar phrase of "a long-headed fellow" means a clever man. Plutarch says, that Pericles had so long a

* The treatise of Hippocrates on this point is unrivalled, for, besides its accurate description of the complaint, it is narrated with the freshness of a recent production. Hippoc.: opera omnia, Græc. et Lat. Lugduni. Batav. apud Graasbechios, 1665. De articulis xxxvi. xxxviii. xxxix. xliv. l. Vectiarius, xxxi.

† Phrenology, in connexion with Physiognomy. Spurzheim. Part I. London, 1826. Plate xxxii. Fig. 1. M. T. Cicero. There is an air of exaggeration in this work, which compels us to receive it with caution. Compare the caricature of P. Gregory VII. with the impartial history of that pontiff recently published by Delécluze. Paris, 1844.

head that he was ashamed of it, and in his statues was always represented with a helmet, on purpose to hide this supposed deformity. Had he enjoyed the advantage of living after the publication of Mr. Solly's present work, the intellectual vanity of the great Athenian might have been induced to hail it as a distinguished beauty.*

No one knows at what conclusions he may arrive by dint of constant observation and study. A British military officer, Major Rawlinson, in an insulated post at Kermanshah, *amuses* himself by first studying, then deciphering, and at last actually reading off the arrow-headed, or trilingual inscriptions of Hamâdan or Behistân, relating to generations of the Persian monarchy long since extinct, whereby our old schoolfellows, Xerxes and Darius Hystaspes, are positively brought to life again, and once more made to speak their mandates for themselves.† And so, likewise, the investigations of Rosselini have entirely cleared up dubious points in chronology, merely by studying *illegible* monuments, and deciphering the scrolls or *scrawls* of hieroglyphical inscriptions. Shishak and Rehoboam are no longer discernible only through the sombre vista of the history of the kings of Judah, but are, like Xerxes and Hystaspes, recovered from the dust of ages, re-clad in the habiliments of state, and associated among ourselves as moving personages on the stage of life.‡ The progress of genius, like that of a great river, is slow, gradual, and expansive, gathering contributions from every lesser source as it passes along, till at last, with its tide of borrowed waters, it breaks forth at its embouchure into the ocean, a mighty flood, apparently all its own. We are none of us either great or learned of ourselves ; for we are only learned and only great by means of those who have gone before us, and in exact proportion to the diligence with which we have followed in their steps, and learnt from their examples.

* Langhorne's Plutarch. Article, Pericles. Note *in loco*. Pericles was generally known by the sobriquet of the Macrocephalus.

† Journal, Asiatic Society, vol. x. part i.

‡ Connexion between Science and Revealed Religion, by Dr. Wiseman. 2nd Edit. 1842. Lecture IX.

